# What Happens When Individuals Answer Questionnaires in Two Different Languages

**DOI:** 10.3389/fpsyg.2021.688397

**Published:** 2021-06-23

**Authors:** Clara Paz, Carlos Hermosa-Bosano, Chris Evans

**Affiliations:** ^1^Escuela de Psicología, Universidad de Las Américas, Quito, Ecuador; ^2^Department of Psychology, The University of Sheffield, Sheffield, United Kingdom

**Keywords:** translation, CORE-OM, SOS-10, outcomes measures, psychological interventions, score comparability, cultural adaptation

## Abstract

The aim of the present study was to compare scores from the English and the Spanish versions of two well-known measures of psychological distress using a within-subject approach. This method involved bilingual participants completing both measures in four conditions. For two groups of people, measures were offered in the same language both times and for the other two groups, each language version was offered, the order differing between the groups. The measures were the Clinical Outcomes in Routine Evaluation-Outcome Measure and the Schwartz Outcome Scale-10, both originally created in English and then translated to Spanish. In total, 109 bilingual participants (69.7% women) completed the measures in two occasions and were randomly allocated to the four conditions (English-English, English-Spanish, Spanish-English and Spanish-Spanish). Linear mixed effects models were performed to provide a formal null hypothesis test of the effect of language, order of completion and their interaction for each measure. The results indicate that for the total score of the Clinical Outcomes in Routine Evaluation-Outcome Measure just language had a significant effect, but no significant effects were found for completion order or the language by order interaction. For the Schwartz Outcome Scale-10 scores, none of these effects were statistically significant. This method offers some clear advantages over the more prevalent psychometric methods of testing score comparability across measure translations.

## Introduction

Translation of psychological measures is a common practice worldwide and there is steadily growing interest in comparing findings across countries and languages. More than 30 guidelines have been created to guide the translation process as well as the cultural adaptation of these measures; however, there is no consensus of which one is the best methodology (Epstein et al., 2015). These procedures all aim to attain equivalence between the original and the translated version of the instrument, but they vary in how much they acknowledge that perfect equivalence is an ideal that is not ensured by any translation method nor even easy to fully define. Despite these challenges, clearly, it is not possible to compare data across translations of measures without some empirical exploration of score comparability. Generally, this is explored using between-subjects approaches that compare the scores given to the measure by persons from different populations looking for “measurement invariance,” a statistical property that indicates that the same construct is measured across samples (Vandenberg and Lance, 2000; Byrne and Watkins, 2003; Milfont and Fischer, 2010). Measurement invariance is usually tested within either Classical Test Theory (CTT), typically through Confirmatory Factor Analysis (CFA); or within Item Response Theory (IRT) methods. These psychometric approaches are sophisticated and, when their assumptions are met, they offer power to detect forms of non-equivalence. However, with two different models, these methods test the covariance of items *across the individuals* between the language samples. This is highly appropriate for measures largely designed to compare individuals' scores at a single completion, e.g., to determine school or university entry or to measure personality traits. However, this is tangential to the aims of measures of within individual change, i.e., to the typical aim of measures used in psychotherapy to assess change over time (Tarescavage and Ben-Porath, 2014). For such measures these forms of measurement invariance are rarely found, even within one language (Kim et al., 2010; Fried et al., 2016). In addition, such measures cannot be used for single item measures such as visual analog scales as such measures have no item covariance to explore. This situation creates a need for other approaches to explore the equivalence of measures across translations.

The aim of the present study was to compare scores from the English and the Spanish versions of two well-known measures of psychological distress using a within-subject approach, a rarely used approach (Spector et al., 2015). One variant of this approach is to offer each language version of the questionnaire to the same group of individuals on two occasions, perhaps randomizing their order (Rivas-Vazquez et al., 2001; Chen and Bond, 2010; Chen et al., 2014; Zavala-Rojas, 2018; Rezapour and Zanjirani, 2020). However, another variant throws more light on language and order effects by using four groups, again with two occasions. For two of the four groups measures are offered in the same language on each occasion; for the other two groups, each language is offered, the order differing between groups. Such studies aiming to estimate any language effect are compromised by the test-retest effect: the very common finding that when mental health measures are completed twice in non-help-seeking samples there is often a mean shift between occasions (see Durham et al., 2002, for a review). The four-group design disaggregates the test-retest effect from any effect of language allowing that test-retest mean change might interact with language, something that cannot be done in the two group method. Studies using the two group method, specifically to test measures used to assess change in psychotherapy, are scarce. The only study that we found is the one conducted by Wiebe and Penley (2005) using the Beck Depression Inventory, and there, no significant language effects on mean scores were founded. However, studies using that approach for personality measures have found language effects for some traits (Chen and Bond, 2010; Chen et al., 2014; Rezapour and Zanjirani, 2020), suggesting that language might activate cognitive styles and behavioral expressions which are linked to the specific linguistic-social context in which the language was learned or in which it is most commonly used. In relation to time, the study conducted by Wiebe and Penley (2005) reported that the scores were lower in the second completion for all groups, thus indicating that time produced an effect. This study reports the use of the four group method with the Clinical Outcomes in Routine Evaluation-Outcome Measure (CORE-OM; Evans et al., 2000, 2002) and the Schwartz Outcome Scale-10 (SOS-10; Blais et al., 1999), both originally created in English and then translated to Spanish. Both measures are widely used in various languages to track outcomes and change in psychological distress when applying psychological interventions. Also, both measures can be used free of charge which contributes to their growing use in Latin American countries in recent years (Paz et al., 2020a,b). These factors led us to choose these measures to test our method and add to the literature about them in Latin America.

## Methods

### Participants

Participants were bilingual (Spanish-English and English-Spanish) adults, living in Ecuador, a predominantly Spanish-speaking country, who have either completed an International Baccalaureate or have obtained an English proficiency certificate. Participants were recruited by means of posts on alumni social media pages of high schools which offer International Baccalaureate with English as the main language. Also, we asked institutions that offer English lessons to distribute the invitation to participate in the study to people who had attained an English proficiency certificate. Participation in the study was entirely voluntary with no monetary incentives or compensation for the participants.

In total, 167 persons completed the measures on the first occasion, 110 (65.9%) were women and 57 (34.1%) were men. The mean age was 26.41 (*SD* = 7.78) and the age range varied from 18 to 58. Of the 167 participants, 155 described themselves as bilingual and met the eligibility criteria, for four of them English was the native language. On the second occasion measures were completed by 109 participants, 70.3% of those who completed the measures on the first occasion an met elegibility criteria. Of these, 76 (69.7%) were women and 33 (30.3%) were men. The mean age was 26.36 (*SD* = 7.26) with range from 18 to 50. No significant effects of gender [*χ*^2^(1, *N* = 155) = 1.30, *p* = 0.253] or age [*t* (77) = 0.11, *p* = 0.91, *d* = 0.02] were found comparing those who only completed the measures on the first occasion with those completing the measurements on both occasions. More fundamentally, the language in which the questionnaire was first presented was not statistically significantly related to non-completion on second occasion: χ^2^ (1, *N* = 155) = 3.31, *p* = 0.07. The mean number of days between first and second occasion was 20.1 (*SD* = 6.68) ranging from 14 to 40 days. The breakdown into groups of those who participated on both occasions was English-English = 20, English-Spanish = 32, Spanish-English = 32 and Spanish-Spanish = 25.

### Measures

#### CORE-OM

This instrument is a self-report questionnaire containing 34 items that assess general psychological state (Evans et al., 2000, 2002). The Spanish translation (Feixas et al., 2012) was conducted in Spain (Trujillo et al., 2016) and their psychometric properties of the scores were good and similar to those reported for the original version. The psychometric properties of the Spanish version offered to an Ecuadorian sample (Paz et al., 2020b) indicated that these properties are similar to those reported for the scores of the original version in United Kingdom (Evans et al., 2002) and to the Spanish version in Spain. In the present study, the Cronbach alpha of the English version was 0.96, 95% CI [0.93, 0.98] and that of the Spanish version was 0.93, 95% CI [0.90, 0.95].

#### SOS-10

This instrument is a 10-item self-report measure of well-being (Blais et al., 1999). This measure was translated to Spanish in the United States with a group of bilingual individuals (Rivas-Vazquez et al., 2001), and the exploration of the psychometric properties of the scores obtained in that study indicate that they are good and comparable with those found for the original version in English (Blais et al., 1999). The psychometric properties of this measure have been also tested in Ecuador (Paz et al., 2020a). Results from this study indicate that the properties are similar to those found for the original and the Spanish translations (both conducted in United States). In the present study Cronbach alpha was 0.94, 95% CI [0.90, 0.96] for the English version and 0.93, 95% CI [0.89, 0.95] for the Spanish version.

### Procedures

The sample size was calculated by simulation which showed that a sample size of at least 100 participants, assuming a minimal test-retest stability of 0.6 would give 95% effect size confidence intervals of +/−0.16 for the effect of language assuming equal sized groups.

Participants were contacted through social media and invited to participate in an online anonymous study conducted using LimeSurvey (LimeSurvey Project Team, 2012). Participants who gave informed consent completed a brief sociodemographic questionnaire and were then randomly allocated to complete the measures (CORE-OM and SOS-10) either in Spanish or in English. Then, 14 days later participants were invited to complete the measures again and they were randomly allocated again to complete the measures either in Spanish or in English. Hence four conditions of the presentation of the measures were created: (1) English on both occasions (EE), (2) English the first occasion and Spanish on the second occasion (ES), (3) Spanish the first occasion and English on the second occasion (SE), and (4) Spanish on both occasions (SS). If participants did not complete measures on the second occasion, they were reminded weekly, until the termination of data collection. The random allocation aimed to balance the groups, but it was recognized that random allocation and attrition would be very unlikely to achieve perfectly balanced group allocation. The survey was set up with all questions mandatory hence there were no missing data. The Ethics Committee of the Universidad de Las Américas, Ecuador approved the study [ID: 2020-0619].

### Data Analysis

Linear mixed effects models were performed to provide a formal null hypothesis test of the effect of language, order of completion and their interaction. Regression models were conducted separately for each measure (CORE-OM and SOS-10). As there can be gender differences in mean scores on such measures, gender was recorded and entered as a simple participant-level co-variable. However, statistically significant gender effects were not expected given the sample size, between groups test and the low expected effect. Age effects are generally very small, and the age range of the participant pool was small; for these reasons it was not treated as a covariate. These analyses were conducted using the nlme: Linear and Non-linear Mixed Effects Models package (Pinheiro et al., 2021) from R statistical software (R Core Team, 2020).

## Results

To test language effects, measurement completion order and their interaction, independent models were performed for each measure. Total scores of each measure were included as the dependent variables and gender, language, completion order and the interaction of language and order of completion were included as fixed effects, while the subjects were placed as random effects for each model.

**Figure 1:**
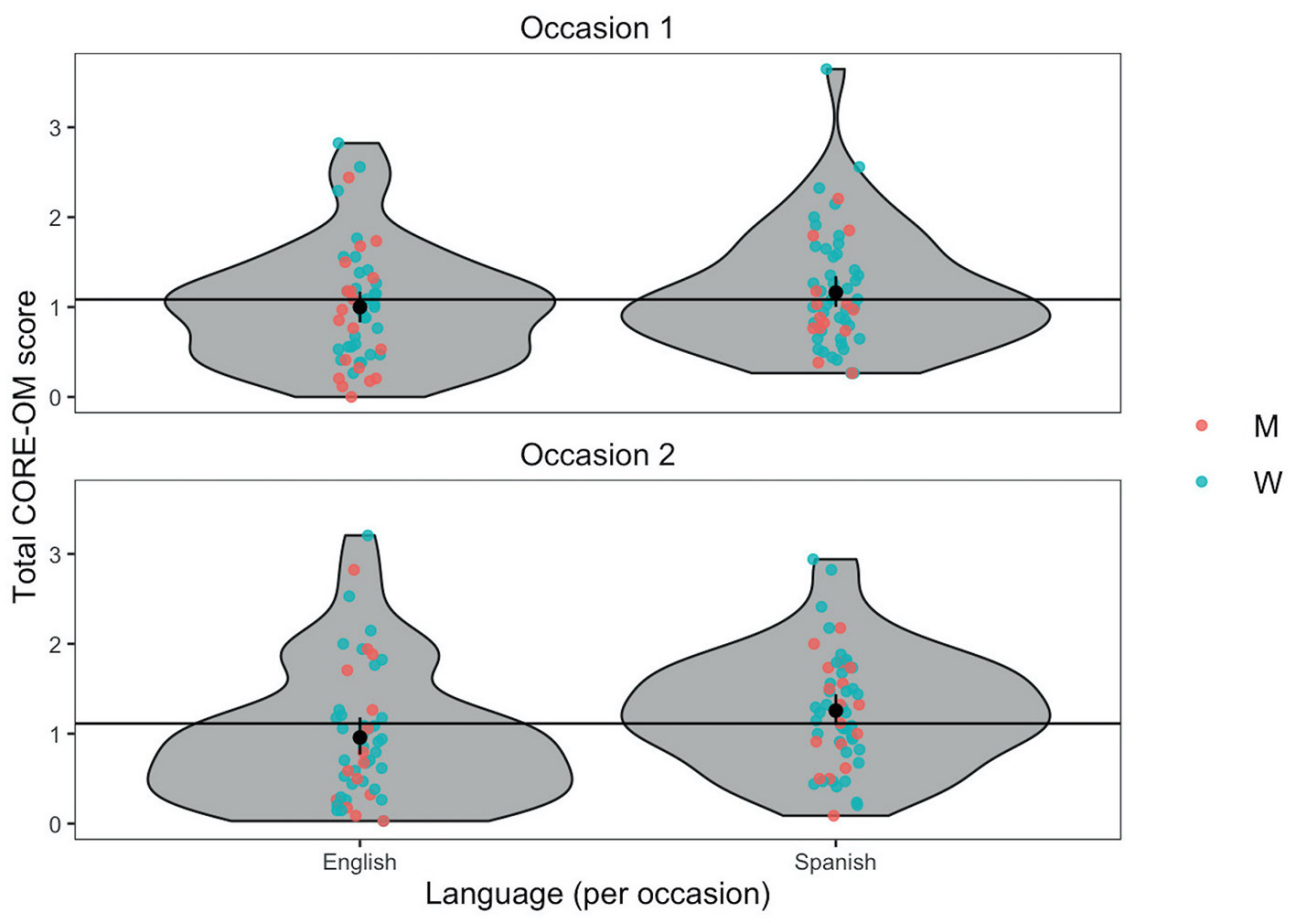
Violin plot of CORE-OM scores by language, gender, and occasion. Jittered points show individual scores by gender, horizontal reference lines are mean scores by occasion, and black points and error bars are language means within occasion with 95% bootstrap confidence interval. Points are jittered horizontally to minimize possible overprinting but not jittered vertically so the scores are accurately represented. W, women; M, men.

For the total score of the CORE-OM, results indicated that neither of completion order (*β* = 0.02, *SE* = 0.06, *df* = 106, *p* = 0.78) nor participants' gender (*β* = −0.11 *SE* = 0.13, *df* = 107, *p* = 0.36) showed statistically significant effects. Language did show a significant effect (*β* = −0.18, *SE* = 0.07, *df* = 106, *p* = 0.02) with no significant interaction with order of completion (*β* = 0.03, *SE* = 0.11, *df* = 106, *p* = 0.82). Figure 1 shows the violin plot of the CORE-OM total scores by language, gender and occasion. In this figure a tendency for total scores on the Spanish version to be higher than on the English version is visible, however the difference is within the precision of estimation of the means as shown by the vertical bootstrap 95% confidence interval lines crossing the shared mean scores for each occasion. Mean differences and effect sizes of the CORE-OM scores by group are presented in Table 1.

**Table 1:** Mean differences and effect sizes for the effect of time and language.

	**CORE-OM**		**SOS-10**	
**Variable**	**Mean difference *M* [95% CI]^**a**^**	**Effect size Hedges' g [95% CI]^**a**^**	**Mean difference *M* [95% CI]^**a**^**	**Effect size Hedges' g [95% CI]^**a**^**
English-English	−0.11 [−0.60, 0.32]	−0.13 [−0.80, 0.40]	1.25 [−7.65, 9.65]	0.09 [−0.53, 0.72]
English-Spanish	−0.17 [−0.45, 0.12]	−0.28 [−0.76, 0.21]	−0.47 [−6.44, 5.40]	−0.04 [−0.53, 0.45]
Spanish-English	0.15 [−0.16, 0.50]	0.22 [−0.24, 0.73]	−1.94 [−7.72, 3.50]	−0.16 [−0.67, 0.31]
Spanish-Spanish	−0.03 [−0.36, 0.30]	−0.04 [−0.64, 0.49]	1.40 [−4.92, 7.64]	0.12 [−0.40, 0.72]

a *Bootstrapped 95% confidence interval*.

**Figure 2:**
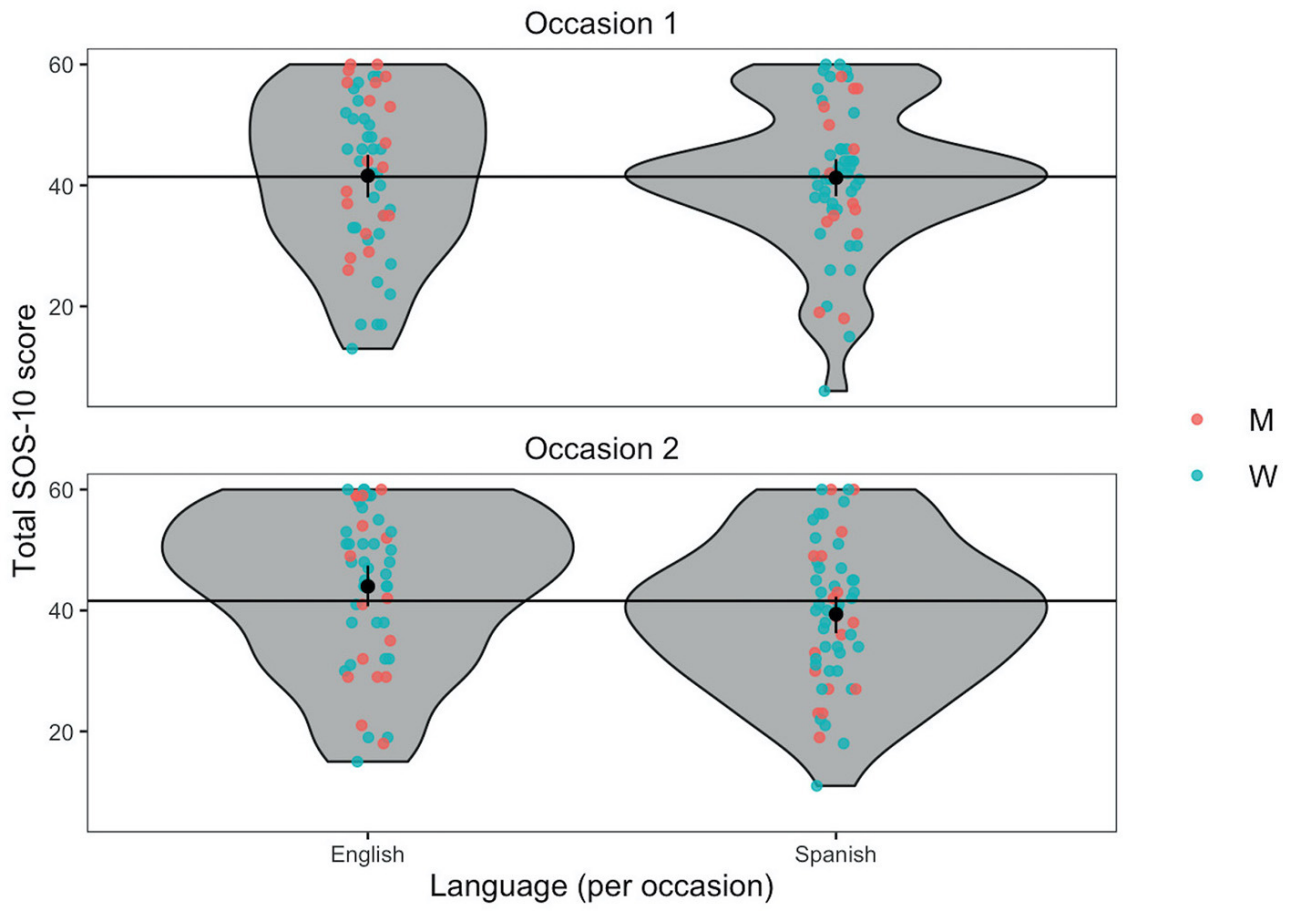
Violin plot of SOS-10 scores by language, gender and occasion. Jittered points show individual scores by gender, horizontal reference lines are mean scores by occasion, and black points and error bars are language means within occasion with 95% bootstrap confidence interval. Points are jittered horizontally to minimize possible overprinting but not jittered vertically, so the scores are accurately represented. W, women; M, men.

For the SOS-10 scores, no significant effects were found for completion order (*β* = −0.51, *SE* = 1.32, *df* = 106, *p* = 0.69), gender (*β* = 0.07, *SE* = 2.45, *df* = 107, *p* = 0.97), language (*β* = 0.34, *SE* = 1.48, *df* = 106, *p* = 0.82), nor for the interaction between the order of completion and the language (*β* = 1.41, *SE* = 2.23, *df* = 106, *p* = 0.52). Figure 2 shows the violin plot of the SOS-10 total scores by language, gender and occasion which shows that scores for the English version tended to be higher than for the Spanish version, but the difference is clearly within the precision of estimation of the means. Mean differences and effect sizes of the SOS-10 scores by group are presented in Table 1.

## Discussion

This is one of the few studies that have empirically tested the effects of language and completion order of two psychological distress measures in a bilingual sample (Spanish and English). The results indicate that for the total score of the CORE-OM, language had a small but statistically significant effect, but neither gender, completion order, nor the language by completion order interaction presented significant effects. For the SOS-10 none of these effects were significant. Then, it seems that Spanish and English versions of the CORE-OM are not perfectly equivalent, but the differences, smaller than one point on a score with range from 0 to 40 and with 95% confidence intervals for the differences also under one score point, were sufficiently small and sufficiently precisely estimated to suggest that language change in a population speaking both English and Spanish is not likely to invalidate the use of change scores nor to rule out comparison of score changes from samples in either language. Detected language effect and possible explanations for their existence might be explored in future studies with this specific and other similar measures.

Language effects have been founded in previous studies using personality measures (Chen and Bond, 2010; Chen et al., 2014; Rezapour and Zanjirani, 2020), but not when using measures of psychological distress (Wiebe and Penley, 2005). The results of our study indicate the presence of this effect in one measure but not in the other, which suggest that further studies have to conducted with this type of measures to arrive to more consistent conclusions. However, there is also evidence that response styles are different in different languages and countries (Harzing, 2006), so that possibly explain the presence of a language effect in our data.

The present study has several limitations. First, this study was conducted completely online due the restrictions imposed by the Coronavirus Disease-19 pandemic during 2020; in Ecuador, paper-and-pencil format remains much commoner than online. However, the pandemic brings is probably changing this so generalizability may be less affected by this in the future. Second, in this study we only considered two occasions for assessment. A common practice in psychological intervention is to apply the same instrument at different moments of the intervention. Whether resources are available, future studies might test more complex designs, including more assessment occasions, and might seek bigger samples.

A more general limitation of this method is that people must have sufficient competence in both languages to participate. In countries where bilingualism is common this may not limit generalizability, however, in countries where bilingualism in the chosen languages is uncommon, as in Ecuador, such a method should be complemented by conventional explorations in unilingual samples. There is psychometric literature suggesting that language in use by bilingual or multilingual people can affect questionnaire responses, and variables such as age of acquisition of the languages, dominance and proficiency can affect reception of, and communicating ideational and emotional material in each language (Paradis, 2008; Pavlenko, 2008). Rather separate from that literature there is largely qualitative literature about the bilingualism in psychological therapies, *inter alia* de Zulueta (1995); Pérez Foster (1996); Dale and Altschuler (1997); Das (2020) and it may be relevant that most of the development of analytic therapies in the late 19th and 20th centuries was led by bilingual or multilingual individuals, often working in contexts in which unilingualism was rare. In our study most of the bilingual participants were using their second language (English for most) in academic contexts which can impact on socialization and integration of affective words (Pavlenko, 2008), words that are commonly present in therapy measures. Clearly we can never know if people with fluency in two languages will experience questionnaires in either language exactly as their peers lacking this fluency. A useful extension of this study might be to evaluate and categorize participants' levels of fluency in the languages which might be used as covariates in the four group design and future studies might ask about proficiency, ages of acquisition and contexts where each language is used. Equally, a qualitative extension exploring the experiences when answering the measures could add to the quantitative findings.

Despite these limitations this study presents a new method to evaluate score comparability for translated mental health measures and we believe the method offers advantages over, and a very useful complement to the more prevalent between groups exploration of measurement invariance in terms of cross-sectional item score covariance. This approach maps more clearly than those methods to the widespread use of such measures to evaluation within-individual change rather than between-individual score comparisons (Newsom, 2015) and can be applied to single item measures.

## Data Availability Statement

The original contributions presented in the study will be publicly available. The data will be found at figshare repository: The datasets presented in this study can be at: figshare (doi: https://doi.org/10.6084/m9.figshare.14336609).

## Ethics Statement

The studies involving human participants were reviewed and approved by the Ethics Committee of the Universidad de Las Américas [ID: 2020-0619]. The participants provided their written informed consent to participate in this study.

## Author Contributions

CP and CE designed the study, performed data analysis, and reported results. CE conducted the power estimation simulations. CP and CH-B implemented the study and collected data. CP, CE, and CH-B participated in writing the article. All authors agree to be accountable for the content of the work.

## Conflict of Interest

The authors declare that the research was conducted in the absence of any commercial or financial relationships that could be construed as a potential conflict of interest.
